# Preservation of Anatomical Specimens with Paraffine

**Published:** 1879-03

**Authors:** 


### PRESERVATION OF ANATOMICAL SPECIMENS WITH PARAFFINE.

M. Frank exhibited to the Biological Society of Paris for M. Fredericy the brain and liver of a dog which were preserved in paroffine. The two specimens were well hardened and had each kept their normal color. M. Fredericy made known his process in 1876 in the Bulletin of the Academy of Sciences of Brussels. He commences by macerating his specimens in pure alcohol for several days and afterwards in spirits of turpentine. After this he immerses them in paraffine heated on a water-bath never above sixty degrees centigrade. In cooling, the paraffine impregnates the specimen which is suspended by a thread. M. Fredericy added that his process is only applicable to small specimens. Human brains are too large to be treated in this way. Small specimens are preserved almost indefinitely. Those presented by M. Frank were prepared three years ago.—*Progres Medical.*

